# Supplementary material for: Marine conservation palaeobiology: What does the late Quaternary fossil record tell us about modern-day extinctions and biodiversity threats?
Source: Camb Prism Extinct. 2023 Nov 8;1:e24. doi: 10.1017/ext.2023.22 (PMC11895752; doi:10.1017/ext.2023.22)
Supplement: Kowalewski et al. supplementary material [file S2755095823000220sup001.docx]

**Supplementary Materials**

**Marine conservation palaeobiology: What does the late Quaternary fossil record tell us about modern-day extinctions and biodiversity threats?**

Michał Kowalewski^1^ 0000-0002-8575-4711, Rafał Nawrot^2^ 0000-0002-5774-7311, Daniele Scarponi^3^ 0000-0001-5914-4947, Adam Tomašových^4^ 0000-0002-0471-9480, and Martin Zuschin^2^ 0000-0002-5235-0198

**Material and Methods**

**Introduction**

To generate **Figure 1** and **Figure 3**, the IUCN Red List data were downloaded for four invertebrate phyla (Arthropoda, Cnidaria, Echinodermata, and Mollusca) on 2/20/2023 (IUCN, 2023a). Figures were generated using a custom-written script in *R* (R Team, 2022) appended here as **Appendix 1**. The IUCN data are summarised in **Appendix 2** and **Appendix 3**. A system assignment of all taxa was verified by D. Scarponi with all organisms assigned to one of the four systems: “Marine”, “Brackish”, “Terrestrial”, and “Freshwater” (an additional column in **Appendix 2**). **Table 1** itemises the list of the extinct marine species included in the IUCN Red List as of 4/26/2023 (IUCN, 2023b).

In the case of organisms living in multiple systems through their life cycle, the dominant system inhabited by adult forms was selected. For some analyses, the conservation status of species was grouped into four broad categories (“Extinct”, “Endangered”, “Low Risk”, and “Unknown”). These coarser categories were derived as follows:

1. “Unknown” – “Data Deficient”;
2. “Extinct” – “Extinct”, “Extinct in the Wild”;
3. “Endangered” – “Critically Endangered”, “Endangered”, “Vulnerable”;
4. “Lower Risk” – “Near Threatened”, “Lower Risk/near threatened”, “Lower Risk/conservation dependent”, “Lower Risk/least concern”, “Least Concern”.

Note here that in IUCN (2012; fig. 1 therein), the multiple ‘Lower Risk’ categories are grouped into two coarser units (“Near Threatened” and "Least Concern”.) However, we retained the fine scale subdivisions to conduct fidelity analysis (fig. 3) at the finest possible resolution.

**Probability of Detecting Extinctions for Different Sampling Coverage**

The probability of detecting at least one extinction is given by:

P = 1 – (1 – X)^n^

Where, X is proportion of extinct species in the system, and n is sample size. Thus, for the three sample systems the values are as follows:

Marine P = 1 – (1 – 0.00122737)^95^ = 0.1101228

Freshwater P = 1 – (1 – 0.01050584)^95^ = 0.6333451

Terrestrial P = 1 – (1 – 0.02477910)^95^ = 0.9077885

For sample size of n = 95 species, almost 5% extinction rate is required to detect at least one extinction with probability of 0.99:

X = 1 – $\sqrt[n]{1-P}$ = 1 – $\sqrt[95]{1-0.99}$ = 0.0473193

P = 1 – (1 – 0.0473193) ^95^ = 0.99

Note that the R script provided in **Appendix 1** provides a resampling simulation that reproduces approximate values for the above calculations using random resampling (the outcomes are similar for resampling conducted with or without replacement).

**Fossilization Potential and Fidelity**

For the analyses presented on Figure 3, the fossilisation potential was scored for all taxa (**Appendix 4**) into three categories: 0 – none or low (organisms with no biomineralised skeleton or with skeletal parts limited to microscopic elements such as spicule or sclerites), 1 – intermediate (organisms with weakly biomineralised skeletons or with multi-elemental skeleton hold by soft tissue and thus prone to quick disarticulation), 2 – high (organisms with heavily biomineralised skeletons including, for example, most mollusks, stony corals, and barnacles). The categories were assigned at the ‘order’ level, based on dominant skeletal type within a given group. Category 2 was used as ‘preservable taxa’ in the analysis presented on **Figure 3**. See **Appendix 1** for coding details.

**References Cited**

IUCN., 2012. IUCN Red List Categories and Criteria: Version 3.1. Second edition. Gland, Switzerland and Cambridge, UK: IUCN. iv + 32pp

IUCN., 2023a. *The IUCN Red List of Threatened Species. Version, 2022-2*. https://www.iucnredlist.org. Accessed on February 20, 2023.

IUCN., 2023b. *The IUCN Red List of Threatened Species. Version, 2022-2*. https://www.iucnredlist.org. Accessed on April 26, 2023.

R Core Team (2022). R: A language and environment for statistical computing. R Foundation for Statistical Computing, Vienna, Austria. URL <https://www.R-project.org/>.

**Appendices**

**Appendix 1.** The custom-written *R* Script to generate Figures 1 and 3. A txt-formatted file. Appended below.

**Appendix 2.** IUCN Red List status of species from four invertebrate phyla (IUCN 2023a). A csv-formatted (zip-compressed) file available on github at <https://github.com/MJKowalewski/extinction-review-appendices/blob/main/appendix%202.zip>.

**Appendix 3.** IUCN Red List of species from four invertebrate phyla (IUCN 2023a). A csv-formatted file available on github at <https://github.com/MJKowalewski/extinction-review-appendices/blob/main/appendix%203.csv>.

**Appendix 4.** A list of orders for four invertebrate groups analyzed in figures 1 and 3, with fossilization potential assigned to each order separately based on predominant skeletal type within that order. A csv-formatted file available on github at <https://github.com/MJKowalewski/extinction-review-appendices/blob/main/appendix%204.csv>.

**Appendix 1**

The custom-written *R* Script to generate Figures 1 and 3. A txt-formatted file. Appended below.

# -----------------------------------------------------------------------------

#

# APPENDIX 1 - R Script

#

# Kowalewski, Michał; Rafał Nawrot; Daniele Scarponi; Adam Tomašových; and

# Martin Zuschin (2023) Marine conservation palaeobiology: What does

# the late Quaternary fossil record tell us about modern-day

# extinctions and biodiversity threats? Cambridge Prisms: Extinction.

#

# Written by: M. Kowalewski, kowalewski@ufl.edu

# Last updated: October 26, 2023

#

# -----------------------------------------------------------------------------

library(vegan)

outPDF <- TRUE # set to 'T' or TRUE to output pdf files with figures

pdf.options(family='Helvetica', pointsize=10)

my.col=c('orange3', 'gray', 'orange', 'red4', 'red1',

'green1','green3', 'yellowgreen', 'yellow2')

# --------------- dataset uploads and preparation -----------------------------

# dataset uploads and preparation

status <- read.csv('appendix 2.csv')

taxa <- read.csv('appendix 3.csv')

table(taxa$phylumName)

all(taxa$scientificName == status$scientificName)

orders <- read.csv('appendix 4.csv')

ords <- as.factor(taxa$orderName)

taxa$taph <- NULL

for (i in levels(ords)) {

taxa$taph[taxa$orderName==i] <- orders$Preservation[orders$Var1==i]

}

table(taxa$orderName, taxa$taph)

# group by system

status$prevalent_system[status$prevalent_system == 'Freshwater (=Inland waters)'] <- 'Freshwater'

status$prevalent_system[status$prevalent_system == 'Terrestrial|Freshwater (=Inland waters)'] <- 'Freshwater'

systems <- sort(table(status$prevalent_system))

status$redlistCategory[status$redlistCategory=='Extinct in the Wild'] <- 'Extinct'

# Groups status categories

status$gstatus <- NULL

status$gstatus[status$redlistCategory=='Data Deficient'] <- 'Unknown'

status$gstatus[status$redlistCategory=='Lower Risk/near threatened'] <- 'Lower Risk'

status$gstatus[status$redlistCategory=='Lower Risk/conservation dependent'] <- 'Lower Risk'

status$gstatus[status$redlistCategory=='Lower Risk/least concern'] <- 'Lower Risk'

status$gstatus[status$redlistCategory=='Least Concern'] <- 'Lower Risk'

status$gstatus[status$redlistCategory=='Extinct'] <- 'Extinct'

status$gstatus[status$redlistCategory=='Critically Endangered'] <- 'Endangered'

status$gstatus[status$redlistCategory=='Endangered'] <- 'Endangered'

status$gstatus[status$redlistCategory=='Near Threatened'] <- 'Lower Risk'

status$gstatus[status$redlistCategory=='Vulnerable'] <- 'Endangered'

table(status$gstatus)

status$gstatus <- factor(status$gstatus,

levels=c('Extinct', 'Endangered', 'Lower Risk', 'Unknown'))

# --------------- FIGURE 1 ----------------------------------------------------

statussystem <- table(status$gstatus, status$prevalent_system)[,c(3,1,2,4)]

nsyst <- colSums(statussystem)

statussystem

# function to sample-standardize frequencies and proportions of IUCN categories for all

# systems standardized to the sample size of the smallest (brackish) system (n=95) [FIGURE 1C]

stand.brack.f <- function(k) {

stand.brack <- NULL

dist.syst <- as.factor(rep(1:4, statussystem[,k]))

for (i in 1:10000) stand.brack <- rbind(stand.brack, table(sample(dist.syst, nsyst[2], replace=T)))

apply(stand.brack, 2, mean)/nsyst[2]

}

stand.brack.f2 <- function(k) {

stand.brack <- NULL

dist.syst <- as.factor(rep(1:4, statussystem[,k]))

for (i in 1:10000) stand.brack <- rbind(stand.brack, table(sample(dist.syst, nsyst[2])))

apply(stand.brack, 2, function(x) sum(x>0))/10000

}

pstasys <- t(t(statussystem)/nsyst)

pstasys2 <- t(t(statussystem^(1/4))/colSums(statussystem^(1/4))) # not used

pstasys3 <- cbind(stand.brack.f(k=1), pstasys[,2], stand.brack.f(k=3), stand.brack.f(k=4))

pstasys4 <- cbind(stand.brack.f2(k=1), c(NA,NA,NA,NA), stand.brack.f2(k=3), stand.brack.f2(k=4))

colnames(pstasys3) <- colnames(pstasys) # sample-standardized data

colnames(pstasys4) <- colnames(pstasys) # sample-standardized data

1 - (1 - pstasys[1,1])^95

1 - (1 - pstasys[1,3])^95

1 - (1 - pstasys[1,4])^95

1 - (1-0.99)^(1/95)

1 - (1 - 0.0473193)^95

status.col <- c('red3', 'orange', 'green3', 'gray')

if (outPDF) pdf('Marine vs Freshwater.pdf', height=7, width=5)

tempar <- par(mfrow=c(3,1), mar=c(3,4,0,1), oma=c(3,2,2,1))

barplot(nsyst, col='black', ylim=c(0,15000), las=1,

cex.axis=1, names.arg=c('', '', '', ''))

mtext(side=2, line=3.5, 'number of species')

mtext(side=3, line=-1, adj=1, 'a', xpd=NA, cex=1.25)

text(c(0.7, 2, 3.1, 4.3), nsyst+800, nsyst, cex=1)

barplot(pstasys, col=status.col, las=1,

cex.axis=1, border=status.col, names.arg=c('', '', '', ''))

mtext(side=2, line=2.5, 'proportion of species')

mtext(side=3, line=0.5, adj=1, 'b', xpd=NA, cex=1.25)

legend(0, 1.15, xpd=NA, rownames(statussystem), bty='n',

horiz=T, fill=status.col, cex=1.25, pt.cex=3)

barplot(pstasys4, beside=T, col=status.col, las=1,

cex.axis=1, cex.names=1.4, border=status.col)

mtext(side=2, line=2.5, 'detection probability')

mtext(side=3, line=0.5, adj=1, 'c', xpd=NA, cex=1.25)

mtext(side=1, line=-2, adj=0.375, 'NA', cex=1.5)

par(tempar)

if (outPDF) dev.off()

# write.csv(table(paste(taxa$orderName, taxa$className)), 'ordersclass.csv')

# --------------- FIGURE 3 ----------------------------------------------------

# --------------- Preservational potential by system --------------------------

taphall <- table(taxa$taph, status$prevalent_system)

taphall2 <- t(t(taphall)/colSums(taphall))

taxa$taph <- as.factor(taxa$taph)

status.m <- status[status$prevalent_system == 'Marine',]

taxa.m <- taxa[status$prevalent_system == 'Marine',]

taphmar <- table(taxa.m$taph, status.m$redlistCategory)

status.tr <- status[status$prevalent_system == 'Brackish',]

taxa.tr <- taxa[status$prevalent_system == 'Brackish',]

taphtrans <- table(taxa.tr$taph, status.tr$redlistCategory)

status.fresh <- status[status$prevalent_system == 'Freshwater',]

taxa.fresh <- taxa[status$prevalent_system == 'Freshwater',]

taphfresh <- table(taxa.fresh$taph, status.fresh$redlistCategory)

status.terr <- status[status$prevalent_system == 'Terrestrial',]

taxa.terr <- taxa[status$prevalent_system == 'Terrestrial',]

taphterr <- table(taxa.terr$taph, status.terr$redlistCategory)

taphlist <- list(taphmar, taphtrans, taphfresh, taphterr)

my.col=c('orange3', 'gray', 'orange', 'red4', 'red1',

'green1','green3', 'yellowgreen', 'yellow2', 'yellow')

if (outPDF) pdf('RED LIST FIDELITY PLOT.pdf', height=5, width=7.5)

tempar <- par(mfcol=c(2,4), mar=c(3,3,2,3), oma=c(1,1,2,1))

barplot(taphall2[,c(3,1,2,4)], col=c('white', 'gray70', 'black'), cex.names=1,

width=colSums(taphall)[c(3,1,2,4)]^(1/2), ylim=c(0,1), axes=F, axisnames=F)

axis(2, las=1)

text(c(40,90,160,290), rep(-0.1, 4), colnames(taphall2)[c(3,1,2,4)], cex=1, srt=45, xpd=NA)

mtext(side=3, line=1.5, xpd=NA, 'a', las=1, adj=-0.1, cex=1.2)

mtext(side=2, line=4, 'proportion of species', cex=0.8)

legend(-2, 1.1, horiz=T, x.intersp = 0.3, cex=0.9,

c('none or low', 'intermediate', 'high'),

fill=c('white', 'gray70', 'black'), xpd=NA, bty='n')

plot(0, 0, xlab='', ylab='', axes=F, xlim=c(-1,1), ylim=c(-1,1), type='n')

legend(-1.8, 0.65, pch=22, bty='n', pt.bg=my.col[c(4,5,1,3,10,9,8,7,6,2)],

colnames(taphlist[[1]])[c(4,1,3,10,9,8,6,7,5,2)], cex=1.2, pt.cex=2, xpd=NA)

for (i in c(1,3,4)) {

colnames(taphlist[[i]])

xvar <- decostand(taphlist[[i]][3,], 'log')

yvar <- decostand(colSums(taphlist[[i]]), 'log')

xvar <- xvar[c(4,1,3,10,9,8,6,7,5,2)]

yvar <- yvar[c(4,1,3,10,9,8,6,7,5,2)]

my.col2 <- my.col[c(4,5,1,3,10,9,8,7,6,2)]

colnm <- colnames(taphlist[[i]])[c(4,1,3,10,9,8,6,7,5,2)]

colnm[colnm=='Lower Risk/conservation dependent'] <- 'Lower Risk/Consv.Depend.'

barplot(as.vector(yvar), col=my.col2, axes=F)

axis(2, labels=c(0,5,50,500,2000), at=decostand(rbind(0,5,50,500,2000),'log'), las=1)

mtext(side=3, line=1.5, adj=1, 'all species', cex=0.7)

if (i == 1) mtext(side=3, line=0.5, adj=1, 'marine systems', cex=0.7)

if (i == 3) mtext(side=3, line=0.5, adj=1, 'freshwater systems', cex=0.7)

if (i == 4) mtext(side=3, line=0.5, adj=1, 'terrestrial systems', cex=0.7)

if (i==1) panelnum <- c('b', 'c'); if (i==3) panelnum <- c('d', 'e'); if (i==4) panelnum <- c('f', 'g')

mtext(side=3, line=1.5, xpd=NA, panelnum[1], las=1, adj=-0.1, cex=1.2)

barplot(as.vector(xvar), col=my.col2, axes=F)

mtext(side=3, line=1.5, adj=1, 'preservable species', cex=0.7)

if (i == 1) mtext(side=3, line=0.5, adj=1, 'marine systems', cex=0.7)

if (i == 3) mtext(side=3, line=0.5, adj=1, 'freshwater systems', cex=0.7)

if (i == 4) mtext(side=3, line=0.5, adj=1, 'terrestrial systems', cex=0.7)

axis(2, labels=c(0,5,50,500,2000), at=decostand(rbind(0,5,50,500,2000),'log'), las=1)

mtext(side=3, line=1.5, xpd=NA, panelnum[2], las=1, adj=-0.1, cex=1.2)

}

par(tempar)

if (outPDF) dev.off()

if (outPDF) pdf('RED LIST FIDELITY PLOT.pdf', height=7, width=5.7)

tempar <- par(mfrow=c(3,2), mar=c(3,0,0,4), oma=c(3,9,3,0))

taphlist[[i]]

taphlist[[i]][3,]

barplot(taphall2[,c(3,1,2,4)], col=c('white', 'gray70', 'black'), cex.names=0.9,

width=colSums(taphall)[c(3,1,2,4)]^(1/2), ylim=c(0,1), axes=F, las=1, axisnames=F)

axis(2, las=1)

text(c(40,90,160,290), rep(-0.1, 4), colnames(taphall2)[c(3,1,2,4)], cex=0.8, xpd=NA)

mtext(side=3, line=-1, xpd=NA, 'a', las=1, adj=1.07, cex=1.2)

mtext(side=2, line=4, 'proportion of species', cex=0.8)

legend(0, 1.18, horiz=T, x.intersp = 0.3,

c('none or low', 'intermediate', 'high'),

fill=c('white', 'gray70', 'black'), xpd=NA, bty='n')

plot(0, 0, xlab='', ylab='', axes=F, xlim=c(-1,1), ylim=c(-1,1), type='n')

legend(-0.7, 1.2, pch=c(3,25,25,25,21,21,21,21,21,23), bty='n', pt.bg=my.col[c(4,5,1,3,10,9,8,7,6,2)],

colnames(taphlist[[1]])[c(4,1,3,10,9,8,6,7,5,2)], cex=1.2, pt.cex=1.5, xpd=NA)

points(c(-0.8,-0.8), c(0.9, 0.5), type='l', lend=3, lwd=5, col='orange')

text(-1, 0.7, 'endangered', srt=90, col='orange')

points(c(-0.8,-0.8), c(0.45, -0.3), type='l', lend=3, lwd=5, col='green3')

text(-1, 0, 'lower risk', srt=90, col='green3')

for (i in 1:4) {

yvar <- decostand(colSums(taphlist[[i]]), 'log')

xvar <- decostand(taphlist[[i]][3,], 'log')

if (i %in% c(1,3,4)) {

xvar <- xvar[c(4,1,3,10,9,8,6,7,5,2)]

yvar <- yvar[c(4,1,3,10,9,8,6,7,5,2)]

my.col2 <- my.col[c(4,5,1,3,10,9,8,7,6,2)]

my.pch=c(3,25,25,25,21,21,21,21,21,23)

}

if (i == 2) {

my.col2 <- my.col[c(2,6,3)]

my.pch <- c(23,21,25)

}

plot(xvar, yvar, axes=F, type='n', xpd=NA, xlab='', ylab='',

xlim=c(0,15), ylim=c(0,15))

abline(a = 0, b = 1, lty=2, col='gray', lwd=2)

points(xvar, yvar, pch=my.pch, bg=adjustcolor(my.col2,0.5), cex=2)

if (i %in% c(3,4)) axis(1, at = log2(c(10, 100, 1000, 10000)+1), labels = c(10, 100, 1000, 10000))

if (i %in% c(1,3)) axis(2, las=1, at = log2(c(10, 100, 1000, 10000)+1), labels = c(10, 100, 1000, 10000))

box()

r2 <- round(cor(xvar, yvar)^2,3)

mtext(side=3, adj=0.1, line=-2, bquote(italic(r)^2 == .(r2)), cex=0.75)

mtext(side=3, line=-1, xpd=NA, letters[i+1], las=1, adj=1.07, cex=1.2)

if (i %in% c(3,4)) mtext(side=1, line=3, 'number of preservable species', cex=0.8)

if (i %in% c(1,3)) mtext(side=2, line=4, 'total number of species', cex=0.8)

}

par(tempar)

if (outPDF) dev.off()

# --------------- FIGURE S1 ---------------------------------------------------

if (outPDF) pdf('rsquare resampling model.pdf', height=6, width=4)

tempar <- par(mfrow=c(4,1), mar=c(3,0,0,4), oma=c(3,9,2,0))

for (k in 1:4) {

xobs <- colSums(taphlist[[k]])

ypred <- taphlist[[k]][3,]

xpred <- colSums(taphlist[[k]][1:2,])

out3 <- NULL

for (j in 1:10000) {

out2 <- NULL

for(i in 1:length(xobs)) {

out1 <- sample(c(0,1), ypred[i],

prob=c((xobs[i] - ypred[i])/xobs[i], ypred[i]/xobs[i]), replace=T)

out2[i] <- sum(out1)

}

out3[j] <- cor(decostand(xobs, 'log'), decostand(out2, 'log'))^2

}

r2 <- cor(decostand(xobs, 'log'), decostand(ypred, 'log'))^2

out4 <- hist(out3, col='black', breaks=seq(0,1,0.001), main='', axes=F)

# arrows(r2, 0.35*max(out4$counts), r2, 0, lwd=2, length=0.05, col='green1')

axis(1)

axis(2, las=1)

mtext(side=3, line=-1, adj=0.02, letters[k])

if (k==4) mtext(side=2, line=3.5, 'number of replications', outer=T)

if (k==4) mtext(side=1, line=1, bquote('coefficient of determination ('~r^2~')'), outer=T)

}

par(tempar)

if (outPDF) dev.off()
